# Development of a gene therapy strategy to target hepatocellular carcinoma based inhibition of protein phosphatase 2A using the α-fetoprotein promoter enhancer and *pgk* promoter: an *in vitro* and *in vivo* study

**DOI:** 10.1186/1471-2407-12-547

**Published:** 2012-11-23

**Authors:** Wei Li, Dao-Ming Li, Kai Chen, Zheng Chen, Yang Zong, Hong Yin, Ze-Kuan Xu, Yi Zhu, Fei-Ran Gong, Min Tao

**Affiliations:** 1 Department of Oncology, the First Affiliated Hospital of Soochow University, Suzhou, 215006, China; 2 Department of General Surgery, the First Affiliated Hospital of Nanjing Medical University, Nanjing, 210029, China; 3 Department of Hematology, the First Affiliated Hospital of Soochow University, Suzhou, 215006, China; 4 Jiangsu Institute of Hematology, the First Affiliated Hospital of Soochow University, Suzhou, 215006, China; 5 Key Laboratory of Thrombosis and Hemostasis of Ministry of Health, the First Affiliated Hospital of Soochow University, Suzhou, 215006, China; 6 Institute of Medical Biotechnology, Soochow University, Suzhou, 215021, China

**Keywords:** Hepatocellular carcinoma, AFP, Pgk, PP2A

## Abstract

**Background:**

Hepatocellular carcinoma (HCC) is one of the leading causes of cancer-related deaths worldwide. Current therapies are insufficient, making HCC an intractable disease. Our previous studies confirmed that inhibition of protein phosphatase 2A (PP2A) may provide a promising therapeutic strategy for cancer. Unfortunately, constitutive expression of PP2A in normal tissues limits the application of PP2A inhibition. Thus, a HCC-specific gene delivery system should be developed. The α-fetoprotein (*AFP*) promoter is commonly used in HCC-specific gene therapy strategies; however, the utility of this approach is limited due to the weak activity of the *AFP* promoter. It has been shown that linking the *AFP* enhancer with the promoter of the non-tissue-specific, human housekeeping phosphoglycerate kinase (*pgk*) gene can generate a strong and HCC-selective promoter.

**Methods:**

We constructed a HCC-specific gene therapy system to target PP2A using the *AFP* enhancer/*pgk* promoter, and evaluated the efficiency and specificity of this system both *in vitro* and *in vivo*.

**Results:**

*AFP* enhancer/*pgk* promoter-driven expression of the dominant negative form of the PP2A catalytic subunit α (DN-PP2Acα) exerted cytotoxic effects against an AFP-positive human hepatoma cell lines (HepG2 and Hep3B), but did not affect AFP-negative human hepatoma cells (SK-HEP-1) or normal human liver cells (L-02). Moreover, *AFP* enhancer/*pgk* promoter driven expression of DN-PP2Acα inhibited the growth of AFP-positive HepG2 tumors in nude mice bearing solid tumor xenografts, but did not affect AFP-negative SK-HEP-1 tumors.

**Conclusions:**

The novel approach of *AFP* enhancer/*pgk* promoter-driven expression of DN-PP2Acα may provide a useful cancer gene therapy strategy to selectively target HCC.

## Background

Hepatocellular carcinoma (HCC) is one of the most prevalent tumor types worldwide, especially in several areas of Asia and Africa [1,2]. HCC leads to approximately 662,000 deaths worldwide every year, and the mortality rate is increasing [3,4]. In spite of improvements in diagnosis and clinical treatment methods, HCC remains an aggressive malignant tumor due to the nonspecific symptoms, invasiveness, resistance to chemotherapy and high rate of tumor recurrence [3]. HCC is closely associated with chronic liver disease, particularly cirrhosis due to hepatitis B virus or hepatitis C virus infection [1,5]. Patients with liver cirrhosis and HCC are often poor candidates for surgery, even if the HCC is detected at an early stage, as they generally lack a hepatic reserve as a result of the coexisting advanced cirrhosis [1]. Therefore, new treatments against this aggressive neoplasm are urgently needed.

Cantharidin, the active constituent of the mylabris Chinese blister beetle, has been used as a traditional Chinese medicine for more than 2000 years and is still used as a folk medicine. Cantharidin has an affinity for the liver [6], and has demonstrated therapeutic effects against HCC in clinical trials without suppressing bone marrow function, even in patients at an advanced stage [6,7]. Cantharidin is a potent and selective inhibitor of protein phosphatase 2A (PP2A). The core enzyme of PP2A consists of a catalytic subunit (PP2Ac) and a regulatory A subunit (PP2Aa). A third regulatory B subunit can be associated with this core structure, and this modulates the substrate specificity of PP2A. At present, two isoforms of the α and β catalytic subunits have been identified [8,9]. In previous studies, we proved that cantharidin repressed cancer cell proliferation and triggered apoptosis in a mechanism dependent on the inhibition of PP2A, suggesting that PP2A inhibition may provide a novel approach for hepatoma therapy [7,10,11]. However, the cytotoxicity of cantharidin in normal hepatic tissue and the urinary system restricts its clinical application [6], indicating that a cancer tissue-specific therapy strategy should be developed for the inhibition of PP2A.

Gene therapy using tumor- or tissue-specific promoter-driven suicide genes, immunosuppressors, antiangiogenic genes or tumor suppressor genes is a promising approach for the treatment of cancer. Expression of the α-fetoprotein (*AFP*) gene is reactivated in HCC cells; however, the therapeutic results of *AFP* promoter-driven gene therapy are unsatisfactory, as the transcriptional activity of this promoter is usually weak. It has been proven that the enhancer and silencer regions located upstream of the *AFP* gene play a critical role in the selective expression of AFP in HCC. Additionally, the *AFP* enhancer fragment may provide HCC-specific activity to the promoter of the non-tissue-specific, housekeeping phosphoglycerate kinase (*pgk*) gene, and this novel strategy may be useful for HCC-specific cancer gene therapy [12].

Therefore, in the present study, we attempted to develop a HCC-specific gene therapy system by expressing a dominant negative mutant form of the PP2A catalytic subunit α (DN-PP2Acα) [13] under direct transcriptional control of the *AFP* enhancer/*pgk* promoter, and investigated the therapeutic effects of this system in HCC *in vitro* and *in vivo*.

## Methods

### Cell lines and culture

The AFP-positive human hepatoma cell lines, HepG2 and Hep3B, the AFP-negative human hepatoma cell line SK-HEP-1, and the normal human liver cell line L-02 were purchased from the American Type Culture Collection (Manassas, VA, USA). The cells were maintained in RPMI-1640 medium (DMEM; Gibco, Grand Island, NY, USA) supplemented with 10% fetal calf serum (FCS; Hyclone, Logan, UT, USA), 100 U/ml penicillin and 100 mg/ml streptomycin. The cultures were incubated at 37°C in a humidified atmosphere containing 5% CO_2_, and passaged every 2–3 days to maintain exponential growth.

### MTT assay

Cellular growth was evaluated using the 3-[4,5-dimethyltiazol-2-yl] 2,5-diphenyl-tetrazolium bromide (MTT) assay [14]. The cells were seeded in 96-well plates at 5×10^3^ cells/well. After treatment, MTT (Sigma, St. Louis, MO, USA) was added to each well at a final concentration of 0.5 mg/ml and incubated at 37°C for 4 h. The media was removed, 200 μl dimethyl sulphoxide (DMSO) was added to each well and the absorbance was measured at 490 nm using a microplate ELISA reader (Bio-Rad Laboratories, Hercules, CA, USA). The inhibition rate was calculated as follows: inhibition rate = [(mean control absorbance-mean experimental absorbance)/mean control absorbance] × 100 (%). The concentration which caused a 50% growth inhibition (IC_50_) was calculated using the modified Kärbers method [15] according to the formula: IC_50_ = lg^− 1^[Xk − i(∑p − 0.5)], where Xk represents the logarithm of the highest drug concentration; i is the ratio of the adjacent concentration; and ΣP is the sum of the percentage growth inhibition at various concentrations. The relative cell viability was calculated as follows: relative cell viability = (mean experimental absorbance/mean control absorbance) × 100 (%).

### Serine/threonine phosphatase assay

PP2A activity was analyzed using the nonradioactive serine/threonine-phosphatase assay kit (Promega, Madison, WI, USA) according to the manufacturer’s protocol. In brief, the cell lysate supernatant was passed twice through a Sephadex G-25 spin column to remove free phosphate, the eluate was placed into 96-well plates, and the assay was performed in the presence of a PP2A-specific serine/threonine phosphopeptide substrate (RRApTVA, in which pT represents phosphothreonine). Molybdate dye solution was added to the wells, incubated for 30 min at room temperature, color development was observed, absorbance was measured at 630 nm, and the amount of phosphate released was calculated using a standard curve. The relative activity of PP2A was calculated according to the following equation: PP2A activity = (mean experimental phosphate amount/mean control phosphate amount) × 100 (%).

### Site-directed mutagenesis

Wild-type PP2A catalytic subunit α (PP2Acα) was cloned as previously described [10]. The dominant negative mutant form of PP2Acα (DN-PP2Acα) was PCR-amplified from wild-type PP2Acα (WT-PP2Acα) using site-directed mutagenesis to mutate Leu 199 to Pro [13]. Site-directed mutagenesis was performed by primed PCR amplification of the plasmid [16]. Plasmid template DNA (10 ng) was added to a PCR cocktail containing PrimerSTAR HS DNA polymerase (TAKARA Biochemicals, Dalian, China) and the mutagenic oligonucleotide primers: sense: 5’-CCAATGTGTGACTTGCCGTGGTCAGATCCAGATG-3’; anti-sense: 5’-CATCTGGATCTGACCACGGCAAGTCACACATTGG-3’. The PCR cycling parameters were 30 s at 95°C, followed by 18 cycles of 30 s at 95°C, 1 min at 55°C and 10 min at 72°C. The reaction was placed on ice for 2 minutes, 1 μl *Dpn* I (10 U/μl, New England Biolabs, Ipswich, Massachusetts, USA) was added, incubated at 37°C overnight to digest the parental (i.e., the non-mutated) plasmid template DNA [17] and the recircularized vector DNA incorporating the desired mutations was transformed into competent DH5α E. coli.

### Western blotting

Total protein was extracted using a lysis buffer containing 50 mM Tris–HCl (pH 7.4), 150 mM NaCl, 1% Triton X-100, 0.1% SDS, 1 mM EDTA and supplemented with protease inhibitors [10 mg/ml leupeptin, 10 mg/ml aprotinin, 10 mg/mL pepstatin A, and 1 mM 4-(2-aminoethyl) benzenesulfonyl fluoride]. The protein extract was loaded, size-fractionated by SDS–polyacrylamide gel electrophoresis and transferred to PVDF membranes (Bio-Rad Laboratories, Hercules, CA, USA). After blocking, the membranes were incubated with primary antibodies at 4°C overnight and protein expression was visualized using horseradish peroxidase-conjugated antibodies and enhanced chemiluminescence (ECL) (Amersham Pharmacia Biotech, Buckinghamshire, UK). β-actin was used as an internal control.

### Luciferase reporter gene assay

The *pgk* promoter [18] was cloned into pGL3-Basic (Promega, Madison, WI, USA) using the *Nhe*I and *Bgl*II restriction enzymes (New England Biolabs, Beverly, MA, USA) to generate the reporter plasmid, pGL3-Basic-pgk. The reporter plasmid, pGL3-Basic-AFpg, containing the *AFP* enhancer and *pgk* promoter was constructed as previously described [12]. In brief, the *AFP* enhancer, including the A and B domains [19], was cloned into pGL3-Basic using the *Kpn*I and *Nhe*I restriction enzymes, then the *pgk* promoter [18] was cloned into the *Nhe*I and *Bgl*II restriction sites. The positive control reporter plasmid, pGL3-Control, which contained the *SV40* promoter and enhancer sequences, and the internal control plasmid, pRL-SV40, containing the Renilla luciferase gene, were obtained from Promega. Cells were seeded in 24-well plates and transiently co-transfected with the reporter plasmids (500 ng/well) and the pRL-SV40 plasmid (100 ng/well) using X-tremeGENE HP DNA Transfection Reagent (Roche, Indianapolis, USA) according to the manufacturer's protocol, and the media was renewed after 8 h. After 24 h, the cells were lysed and luciferase activity was measured using the Dual-Luciferase Reporter Assay System (Promega) according to the manufacturer's recommendations using the TD-20/20 luminometer (Turner Designs, Sunnyvale, CA, USA). The results were expressed as relative luciferase activity (the ratio of firefly luciferase activity to Renilla luciferase activity).

### Preparation of recombinant adenoviruses

The shuttle plasmids were respectively recombined with the backbone vector pAdEasy-1 in BJ5183 bacteria. Adenovirus generation, amplification, and titration were performed as previously described [20] and viral particles were purified using the Virabind adenovirus purification kit (Cell Biolabs, Inc., San Diego, CA, USA).

### Apoptosis and cell cycle distribution analysis

Apoptosis was quantified and the cell cycle was analyzed as described by Nicoletti et al. [21]. Briefly, the cells were fixed in 80% chilled ethanol 48 h after treatment, and then incubated with 0.5% Triton X-100 solution containing 1 mg/ml RNase A at 37°C for 30 min. Propidium iodide (PI; Sigma) was added at a final concentration of 50 μg/ml, incubated for 30 min in the dark, and the cellular DNA content was analyzed using a fluorescence-activated cell sorter (FACS; Becton Dickinson, San Jose, CA, USA) and the data was processed using WinMDI29 software (Becton Dickinson).

### Clone-formation assay

Cells were seeded at a density of 1,000 cells/well in 6-well plates, and treated 12 h later. After 10 days, the cells were stained with 1% methylrosanilinium chloride and the numbers of visible colonies were counted. The relative clone formation ability was calculated as: relative clone formation ability = (mean experimental clone number/mean control clone number) × 100 (%).

### Tumor xenograft model and adenovirus treatment

Six- to eight-week old male BALB/c athymic nude mice were purchased from the Shanghai Experimental Animal Center (Shanghai, China) and inoculated on the flank with 5 × 10^6^ HepG2 or SK-Hep-1 cells. Tumors were allowed to grow to a volume of 100 mm^3^, and the animals were divided into four treatment groups: control vehicle injection (n = 6); Ad-CMV-DN-PP2Acα injection (n = 6); Ad-AFpg-luciferase injection (n = 6) and Ad-AFpg-DN-PP2Acα injection (n = 6). Adenovirus vectors (1 × 10^8^ plaque forming units/100 μl) were injected directly into the tumor foci center on days 0, 2 and 4 of treatment. Tumor length and width were measured with calipers over a period of five weeks. Tumor volume was calculated as (length × width^2^)/2. All animals received humane care according to the Institutional Animal Care and Treatment Committee of Soochow University.

### Statistical analysis

Results were expressed as the mean value ± standard deviation (S.D.). Statistical analysis was performed using unpaired Student’s *t*-tests; *P* values less than 0.05 were considered significant.

## Results

### Inhibition of PP2A represses the growth of HCC cells and normal human liver cells

**Figure 1:**
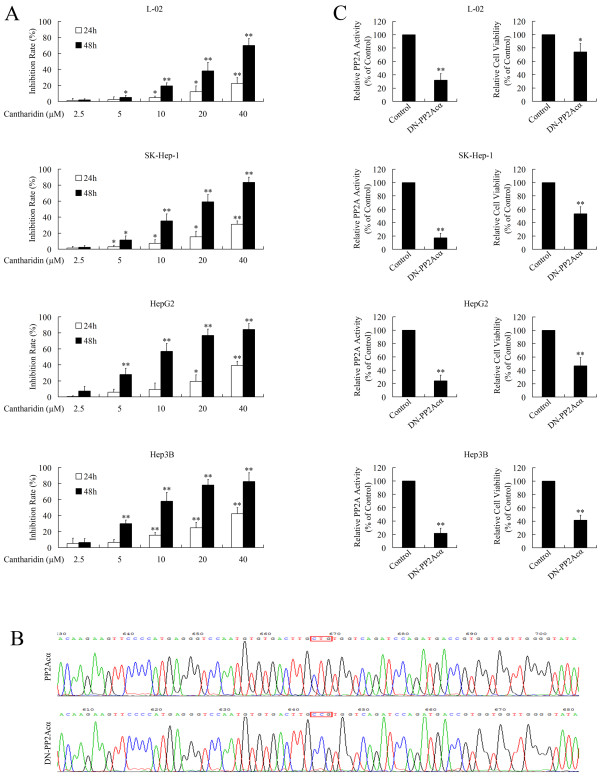
**Inhibition of PP2A induces cytotoxic effects.** (**A**) The MTT assay revealed that the PP2A inhibitor cantharidin repressed cell viability in a dose- and time-dependent manner. (**B**) Sequencing of PP2Acα and DN-PP2Acα. DN-PP2Acα was generated from WT-PP2Acα using site-directed mutagenesis to mutate Leu 199 into Pro. (**C**) The serine/threonine phosphatase assay and MTT assay showed that overexpression of DN-PP2Acα repressed the activity of PP2A and reduced cell viability 48 h after transfection; *P < 0.05 and **P < 0.01 compared to the respective control groups.

The cytotoxic effect of cantharidin against HCC has been widely explored [22,23]. As shown in Figure 1A, cantharidin repressed the growth of normal liver cells and HCC cells in a dose- and time-dependent manner. The IC_50_ of cantharidin in L-02, SK-Hep-1, HepG2 and Hep3B cells at 48 h was 24.97, 15.87, 10.64 and 10.56 μM, respectively. Although cantharidin showed lower cytotoxic effects in normal cells than in cancer cells [23], the potential of cantharidin to harm normal tissues is noteworthy. In fact, the cytotoxicity of cantharidin in normal tissues, especially the hepatic tissue and urinary system, limits the clinical application of cantharidin [6].

Our previous studies confirmed that the mechanism of tumor suppression by cantharidin is mediated via inhibition of PP2A [7,10,11], indicating that PP2A could provide a potential target for the treatment of cancer. To evaluate the cytotoxic effect of specific inhibition of PP2A, a vector carrying dominant negative mutant form of PP2Acα (DN-PP2Acα) was developed. DN-PP2Acα was generated from wild-type PP2Acα (WT-PP2Acα) using site-directed mutagenesis (Figure 1B). As shown in Figure 1C, transfection of pcDNA3.1(+)-DN-PP2Acα repressed the activity of PP2A and inhibited the cell viability of normal liver and HCC cells. However, CMV promoter-driven expression of DN-PP2Acα is not cancer specific, as the CMV promoter drives target gene expression in both normal and cancer cells. Therefore, we designed a tumor specific promoter to achieve HCC-specific inhibition of PP2A.

### AFP-positive-specific expression of DN-PP2Acα using the AFP enhancer/pgk promoter

**Figure 2:**
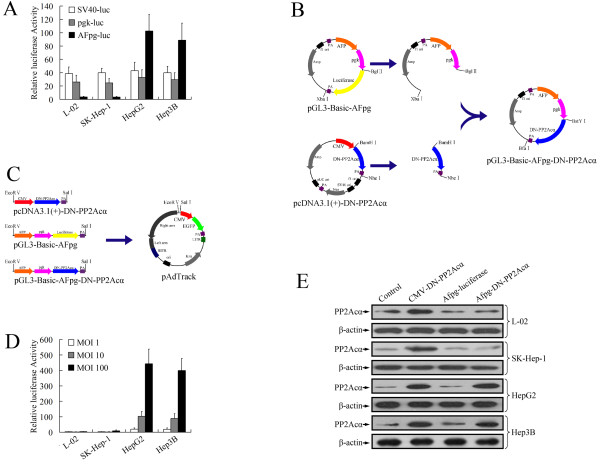
**Specific overexpression of DN-PP2Acα using the *AFP *enhancer/*pgk*promoter (*AFpg*promoter) in AFP-positive HCC cells.** (**A**) The transcriptional activities of the *SV40*, *pgk* and *AFpg* promoters in L-02, SK-Hep-1, HepG2 and Hep3B cells were tested using the luciferase reporter gene assay. (**B**) Construction of the DN-PP2Acα expression vector driven by the *AFpg* promoter. (**C**) Construction of the shuttle plasmids for preparation of recombinant adenoviruses. (**D**) Adenovirus-mediated gene transfer efficiency. Cells were infected with Ad-AFpg-luciferase at various MOI levels. At 24 h post-infection, a luciferase activity assay was performed. (**E**) Western blot analysis of DN-PP2Acα expression after infection of cells with recombinant adenoviruses at a MOI of 100.

It has been demonstrated that linkage of the *AFP* enhancer region to the promoter of the non-tissue-specific housekeeping *pgk* gene may result in increased selectivity for HCC [12]. The luciferase reporter gene assay was used to evaluate the specificity of the *AFP* enhancer/*pgk* promoter (*AFpg* promoter). The transcriptional activity of the *AFpg* promoter was tested in various cell types, including an AFP-positive human hepatoma cell lines (HepG2 and Hep3B), an AFP-negative human hepatoma cell line (SK-Hep-1), and a normal human liver cell line (L-02). Transient transfection experiments demonstrated that luciferase activity was observed in all four cell lines (L-02, SK-Hep-1, HepG2 and Hep3B) with a similar efficiency when transfected with either pGL3-Basic-pgk or pGL3-Control. The activity of the *AFpg* promoter was much lower than the *pgk* promoter in AFP-negative cells (L-02 and SK-Hep-1), but much higher in AFP-positive HepG2 and Hep3B cells (Figure 2A), This indicated that the *AFP* enhancer gave the specificity to the *pgk* promoter and the *AFpg* promoter may be a valuable AFP-positive-specific promoter for gene therapy targeting HCC.

To generate an DN-PP2Acα expression vector driven by the *AFpg* promoter, we replaced the luciferase sequence of pGL3-Basic-AFpg with the coding sequence of pcDNA3.1(+)-DN-PP2Acα. The coding sequence of DN-PP2Acα was PCR-amplified from pcDNA3.1(+)-DN-PP2Acα, digested using *Bam*HI and *Nhe*I, and cloned into the isocaudamer restriction sites, *Bgl*II and *Xba*I, of pGL3-Basic-AFpg. (Figure 2B). Then, the CMV-DN-PP2Acα sequence of pcDNA3.1(+)-DN-PP2Acα, the AFpg-luciferase sequence of pGL3-Basic-AFpg and the AFpg-DN-PP2Acα sequence of pGL3-Basic-AFpg-DN-PP2Acα were cloned separately into pAdTrack using the *Eco*RV and *Sal*I restriction enzymes to generate the adenovirus (Figure 2C).

To determine the transfer efficiency and specificity of adenovirus mediated gene expression driven by the *AFpg* promoter, cells were transduced with Ad-AFpg-luciferase at various multiplicity of infection (MOI) levels. Luciferase activity increased in a dose-dependent manner in two AFP-positive cell lines (HepG2 and Hep3B), but not the AFP-negative cells, L-02 and SK-Hep-1 (Figure 2D).

As expected, the expression levels of PP2Ac showed similar aspects. As shown in Figure 2E, the expression of PP2Ac after transduction with Ad-AFpg-luciferase was not significantly different to the control vehicle group. Transduction with Ad-CMV-DN-PP2Acα induced overexpression of PP2Ac in L-02, SK-Hep-1, HepG2 and Hep3B cells, whereas infection with Ad-AFpg-DN-PP2Acα only led to the overexpression of PP2Ac in AFP-positive HepG2 and Hep3B cells, but not in AFP-negative L-02 or SK-Hep-1 cells. This data indicated that the *AFpg* promoter led to the specific expression of DN-PP2Acα in AFP-positive HCC cells.

### Ad-AFpg-DN-PP2Acα selectively triggers apoptosis and G2/M cell cycle arrest in AFP-positive HCC cells

**Figure 3:**
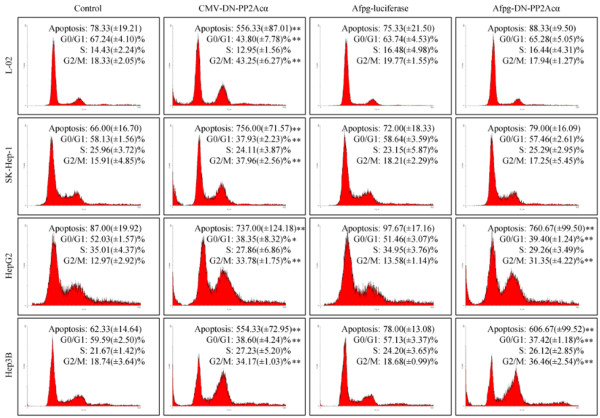
**Expression of DN-PP2Acα driven by the *AFpg *promoter selectively induces apoptosis and alters the cell cycle distribution in AFP-positive HCC cells.** Cells were transducted with recombinant adenoviruses at a MOI of 100. Flow cytometry analysis was performed at 48 h post-infection; *P < 0.05 and **P < 0.01 indicate significant differences compared to the control vehicle group.

In our previous studies, we reported that PP2A inhibitors exerted cytotoxic effects in cancer cells by inducing apoptosis and blocking the cell cycle at the G2/M phase [7,10,11]. In this study, we tested the effect of DN-PP2Acα expression driven by the *AFpg* promoter on apoptosis and cell cycle distribution. As shown in Figure 3, transduction with Ad-CMV-DN-PP2Acα induced apoptosis and G2/M cell cycle arrest in L-02, SK-Hep-1, HepG2 and Hep3B cells. Transduction of Ad-AFpg-luciferase did not significantly alter the level of apoptosis or cell cycle distribution, compared to the control vehicle group. Infection of Ad-AFpg-DN-PP2Acα only triggered apoptosis and G2/M cell cycle arrest in AFP-positive HepG2 and Hep3B cells, but had no effect in AFP-negative L-02 or SK-Hep-1 cells, indicating that specific expression of DN-PP2Acα driven by the *AFpg* promoter selectively induced apoptosis and cell cycle arrest in AFP-positive HCC cells.

### Tissue-specific cytotoxicity of Ad-AFpg-DN-PP2Acα in AFP-positive HCC cells

**Figure 4:**
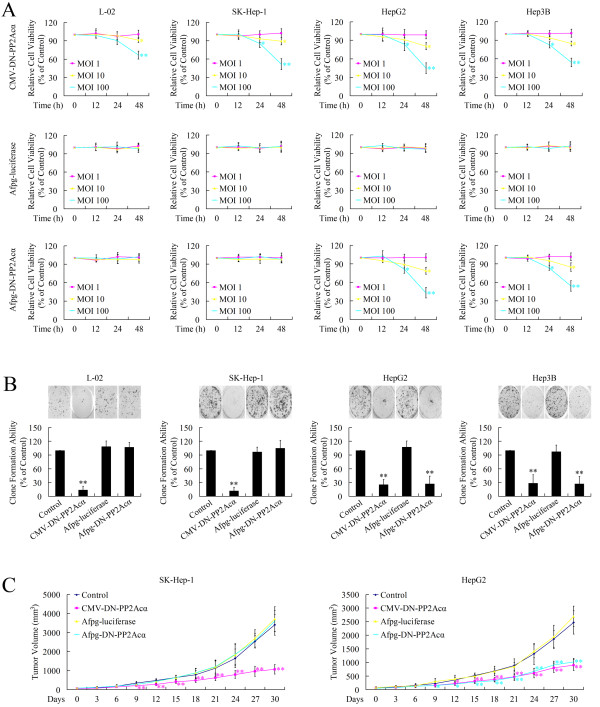
**Expression of DN-PP2Acα driven by the *AFpg *promoter selectively induces cytotoxic effects in AFP-positive HCC cells *in vitro *and *in vivo. *** (**A**) Cells were transducted with recombinant adenoviruses at various MOI levels. At 12 h, 24 h, and 48 h post-infection, the MTT assay was performed. (**B**) Cells were transducted with recombinant adenoviruses at a MOI of 100. After 10 days, the number of visible colonies were counted. (**C**) Effect of AFpg promoter-driven DN-PP2Acα expression on the growth of implanted SK-Hep-1 and HepG2 tumors in athymic mice. Adenoviral gene therapy was initiated when tumors attained a volume of 100 mm^3^. Tumor volume was calculated as (length × width^2^)/2; *P < 0.05 and **P < 0.01 indicate significant differences compared to the control vehicle group.

The effect of DN-PP2Acα expression driven by the *AFpg* promoter on cell growth was further evaluated *in vitro* and *in vivo*. Firstly, *in vitro* studies were performed using the MTT assay and clone formation assay. As shown in Figure 4A, the MTT assay revealed that treatment with Ad-CMV-DN-PP2Acα repressed cell viability in all four cell lines in a time- and dose-dependent manner; however, Ad-AFpg-DN-PP2Acα exerted selective toxicity in AFP-positive HepG2 and Hep3B cells in a time- and dose-dependent manner, but not in AFP-negative L-02 or SK-Hep-1 cells. The clone-formation assay revealed that treatment with Ad-CMV-DN-PP2Acα repressed cell clone-formation ability in all four cell lines; whereas Ad-AFpg-DN-PP2Acα repressed the cell clone-formation ability of AFP-positive HepG2 and Hep3B cells, but not AFP-negative L-02 or SK-Hep-1 cells (Figure 4B).

To extend these findings, *in vivo* studies were performed using SK-Hep-1 and HepG2 xenograft tumor-bearing mice. In mice injected with control vehicle or Ad-AFpg-luciferase, the tumors continued to grow by day 30. Injection of Ad-CMV-DN-PP2Acα significantly diminished the size of both SK-Hep-1 and HepG2 xenograft tumors; however, Ad-AFpg-DN-PP2Acα only inhibited the growth of HepG2 tumor xenografts (Figure 4C). Taken together, these data support the hypothesis that AFpg promoter-driven expression of DN-PP2Acα can induce specific growth inhibition in AFP-positive HCC cells both *in vitro* and *in vivo*.

## Discussion

Gene therapy is a promising approach for the treatment of cancer, and enables the transfer of genetic material to cells to produce a therapeutic effect. A successful gene therapy strategy requires both an effective target gene and a promoter which exhibits high levels of cancer-specific expression.

PP2A (protein phosphatase 2A) is a multimeric serine/threonine phosphatase [24]. In our previous studies, we found that inhibition of PP2A exerted a cytotoxic effect in cancer cells [7,10,11]. Moreover, cantharidin, a potent and selective inhibitor of PP2A, demonstrated promising therapeutic effects against HCC in clinical trials [6,7], suggesting PP2A is a promising target for the treatment of HCC. Unfortunately, the extensive constitutive expression of PP2A in normal tissues, and its complex physiological function obstruct the application of PP2A as a therapeutic target for the treatment of cancer. In clinical trials, cantharidin exerted cytotoxic effects against normal hepatic tissue and the urinary system [6], indicating that the therapeutic inhibition of PP2A must be mediated using a cancer tissue-specific gene delivery system.

To develop a gene therapy system targeting PP2A, we firstly constructed a DN-PP2Acα expression vector driven by the cytomegalovirus (*CMV*) promoter. The *CMV* promoter has been widely used, as it is one of the strongest promoters in mammalian cells. The expression of DN-PP2Acα driven by the *CMV* promoter induced cytotoxicity in HCC cells. The mechanism of DN-PP2Acα induced-cytotoxicity was linked to increased levels of apoptosis and triggering of G2/M cell cycle arrest, as previously described [7,10,11], suggesting that PP2A is a promising target for the treatment of HCC. However, the *CMV* promoter induces target gene expression in both normal cells and cancer cells. As *CMV* promoter-driven expression of DN-PP2Acα induced cytotoxicity in both HCC cells and normal liver cells, cancer-specific delivery and/or gene expression are critical for the safety of gene therapy approaches which aim to inhibit PP2A. To solve this problem, one important approach is to use tumor-specific promoters.

Many cancers often re-express fetal or embryonic genes, and *AFP* gene expression is reactivated in HCC cells. Although the *AFP* promoter is a promising candidate for achieving selective transgene expression in HCC, the weak activity of the *AFP* promoter may limit its utility for gene therapy strategies targeting HCC. It has been proven that the *AFP* enhancer fragment can provide HCC-selective activity to the promoter of the non-tissue-specific, housekeeping gene *pgk*. The *pgk* promoter is recognized as a general, strong promoter and has been used for various gene transfer experiments [25-27]. In this study, addition of the human *AFP* enhancer fragment to the *pgk* promoter provided selectivity to the non-tissue-specific *pgk* promoter in AFP-expressing HCC cells, as previously described [12]. The *AFpg* promoter induced selective cytotoxic effects of DN-PP2Acα in AFP-positive cells. As the *AFpg* promoter has not been evaluated in vivo, we examined the cytotoxic effect of specific expression of DN-PP2Acα, driven by the *AFpg* promoter, in AFP-positive cells using a tumor xenograft model. Ad-AFpg-DN-PP2Acα restrained the tumor growth of AFP-positive xenografts in vivo, but did not affect AFP-negative xenografts.

## Conclusions

In this study, we developed a hepatocellular carcinoma (HCC)-specific gene therapy system by expressing a dominant negative mutant form of the PP2A catalytic subunit under direct transcriptional control of the AFP enhancer/pgk promoter, and investigated the therapeutic effects of this system in HCC *in vitro* and *in vivo*. The data presented indicates that the use of a vector construct targeting PP2A, under the transcriptional control of the *AFP* enhancer fragment and the *pgk* promoter, is a practical and promising strategy to deliver HCC-specific gene therapy.

## Competing interests

The authors declare that they have no competing interests.

## Authors' contributions

WL and DL designed, performed experiments, and participated in drafting the manuscript; KC and ZC participated in plasmids construction; YZ and HY performed flow cytometry assays; ZX and YZ participated in design experiments and discussion of the results; FG and MT conceived of the study and participated in design experiments and coordination, and critically revised the manuscript. The authors read and approved the final manuscript.

## Pre-publication history

The pre-publication history for this paper can be accessed here:

http://www.biomedcentral.com/1471-2407/12/547/prepub
